# Supplementary material for: Coral microbiomes as reservoirs of unknown genomic and biosynthetic diversity
Source: Nature. 2026 Feb 25;652(8110):686–93. doi: 10.1038/s41586-026-10159-6 (PMC13083261; doi:10.1038/s41586-026-10159-6)
Supplement: Supplementary file 1 — Supplementary notes, with sections exploring host transcriptomic responses to microbiome community composition, screening for ELPs in host-associated microbial species, generating S. corallicola and A. pedis genome assemblies and annotations, exploring the effect of the coral host on the growth, transcription profile and actual metabolite production of an acidobacterial strain, functionally characterizing the aci, tha and the clusters, binomial naming of new acidobacterial lineages, stating research (UNCLOS) and CITES permits and acknowledging the local authorities [file 41586_2026_10159_MOESM1_ESM.pdf]

nature portfolio

https://doi.org/10.1038/s41586-026-10159-6

Supplementary information

Coral microbiomes as reservoirs of unknown genomic and biosynthetic diversity

In the format provided by the authors and unedited

Supplementary information

Coral microbiomes as reservoirs of unknown genomic and biosynthetic diversity

Fabienne Wiederkehr[1], Lucas Paoli[1,2,✉], Daniel Richter[3,4], Dora Racunica[3], Hans-Joachim Ruscheweyh[1], Martin Sperfeld[1], James O'Brien[1], Samuel Miravet-Verde[1], Alena B. Streiff[3], Jessica Ransome[3], Clara Chepkirui[3], Taylor Priest[1], Anna Sintsova[1], Guillem Salazar[1,5], Kalia S. I. Bistolas[6], Teresa Sawyer[7], Karine Labadie[8], Kim-Isabelle Mayer[9], Aude Perdereau[8], Maggie M. Reddy[10,11], Clémentine Moulin[12], Emilie Boissin[13], Guillaume Bourdin[14], Juliette Cailliau[12], Guillaume Iwankow[13], Julie Poulain[8,15], Sarah Romac[16], *Tara* Pacific Consortium Coordinators*, Serge Planes[13,15], Denis Allemand[17,18], Sylvain Agostini[19], Chris Bowler[20], Eric Douville[21], Didier Forcioli[17,22], Pierre E. Galand[23], Fabien Lombard[15,24], Pedro H. Oliveira[8], Olivier P. Thomas[10], Rebecca Vega Thurber[6,25], Romain Troublé[12,15], Christian R. Voolstra[9], Patrick Wincker[8,15], Maren Ziegler[26], Jörn Piel[3,✉], Shinichi Sunagawa[1,✉]

1 Department of Biology, Institute of Microbiology and Swiss Institute of Bioinformatics, ETH Zürich, Zürich, Switzerland
2 Global Health Institute, School of Life Sciences, EPFL Lausanne, Lausanne, Switzerland
3 Department of Biology, Institute of Microbiology, ETH Zürich, Zürich, Switzerland
4 Medical Research Council Laboratory of Molecular Biology, Cambridge, UK
5 Institute for Integrative Systems Biology I2SysBio, Universitat de València-CSIC, Paterna, Spain
6 Department of Microbiology, Oregon State University, Corvallis, OR, USA
7 Electron Microscopy Facility, Oregon State University, Corvallis, OR, USA
8 Génomique Métabolique, Genoscope, Institut François Jacob, CEA, CNRS, Université Evry, Université Paris-Saclay, Evry, France
9 Department of Biology, University of Konstanz, Konstanz, Germany
10 School of Biological and Chemical Sciences, Ryan Institute, University of Galway, Galway, Ireland
11 Department of Biological Sciences, University of Cape Town, Cape Town, South Africa
12 Fondation Tara Océan, Base Tara, Paris, France
13 PSL Research University: EPHE-UPVD-CNRS, UAR 3278 CRIOBE, Laboratoire d'Excellence CORAIL, Université de Perpignan, Perpignan, France
14 School of Marine Sciences, University of Maine, Orono, ME, USA
15 Research Federation for the Study of Global Ocean Systems Ecology and Evolution, FR2022/Tara GOSEE, Paris, France
16 Sorbonne Université, CNRS, Station Biologique de Roscoff, AD2M, UMR 7144, ECOMAP, Roscoff, France
17 Laboratoire International Associé Université Côte d'Azur-Centre Scientifique de Monaco (LIA ROPSE), Monaco, Principality of Monaco
18 Centre Scientifique de Monaco, Monaco, Principality of Monaco
19 Shimoda Marine Research Center, University of Tsukuba, Shizuoka, Japan
20 Institut de Biologie de l'École Normale Supérieure (IBENS), École Normale Supérieure, CNRS, INSERM, Université PSL, Paris, France
21 Laboratoire des Sciences du Climat et de l'Environnement (LSCE), CEA, CNRS, UVSQ, Université Paris-Saclay, Gif-sur-Yvette, France
22 Institute for Research on Cancer and Aging, Nice (IRCAN), Université Côte d'Azur, CNRS, INSERM, Nice, France
23 Sorbonne Université, CNRS, Laboratoire d'Ecogéochimie des Environnements Benthiques (LECOB), Observatoire Océanologique de Banyuls, Banyuls-sur-Mer, France
24 Sorbonne Université, Institut de la Mer de Villefranche, Laboratoire d'Océanographie de Villefranche, Villefranche-sur-Mer, France
25 Marine Science Institute, UC Santa Barbara, Santa Barbara, CA, USA
26 Department of Holobiont Biology, Justus Liebig University Giessen, 35392 Giessen, Germany

These authors contributed equally: Fabienne Wiederkehr, Lucas Paoli, Daniel Richter
Correspondence to Lucas Paoli (lucas.paoli@epfl.ch), Jörn Piel (jpiel@ethz.ch), or Shinichi Sunagawa (ssunagawa@ethz.ch)

Supplementary notes

Exploring host-association of BGC-rich microbial species

Coral host transcriptomics and generalised dissimilarity models

We leveraged previously sequenced transcriptomic samples from the coral host *Pocillopora*[152] to explore host transcriptomic responses to microbiome community composition (16S amplicons)[134] along with algal symbiont community composition (ITS2 amplicons)[152], environmental (pH, temperature, oxygen concentration, and nutrients)[54], host phylogenetic distance[153], and host biomarker data[154]. Distances based on normalised transcript-per-million were used as the response variable in a Generalised Dissimilarity Model (GDM)[155].

Firstly, several predictor variables were pre-processed to reduce dimensionality. Community compositions were rarefied using rtk (v0.93.2) to 20,000 reads for 16S amplicons and 4,000 reads for ITS2 amplicons. 16S gene and ITS2 sequences were subsequently aligned against the SILVA SSU reference tree (NR99; release 138) using SINA (v1.6.0). The alignment was processed using MOTHUR (v1.41.0) (filter.seqs) and used to build a phylogenetic tree with FastTree (v2.1.11) (options *-gtr -nt -fastest -mlnni 4*). The rarefied abundances (after excluding ASVs with total abundances below 20 and 4 for microbiome and symbiome data, respectively) together with the phylogenies were then used to reduce the dimensionality of both datasets by grouping 16S and ITS2 ASVs into 116 bins with PhyCA[156]. Environmental and biomarker dimensions were reduced by selecting the columns listed in Supplementary Table 8.

We subsequently computed the model using the R package gdm (v1.5)[155], with host transcriptomic distances as the response variable and the other variables (after range normalisation) as predictors, across the 28 samples for which all data types were available (Supplementary Table 8). In addition, a simpler GDM was built using host transcriptomic distances as the response variable and all Acidobacteriota ASVs with total rarefied abundance greater than five across the 57 samples where both data types were available (Supplementary Table 8). Predictor importance was computed with the gmd.varImp function using 100 permutations.

We found the most important predictor to be a microbiome cluster exclusively containing Acidobacteriota spp. (Extended Data Fig. 3b; Supplementary Table 8). Furthermore, when assessing individual abundances of Acidobacteriota spp. (the most BGC-rich bacteria identified in this study; Fig. 4a) for their predictability of host transcriptomic dissimilarities, several candidate superproducer genera and candidate superproducer-containing lineages stood out as the explanatory features (Extended Data Fig. 3c).

Screening for ELPs in host-associated microbial species

We found BGC-rich microbial species in general, and Acidobacteriota spp. in particular, to be more enriched in ELPs (Extended Data Fig. 2b) than other previously reported coral symbionts such as *Endozoicomonas* spp.[157,158].

Improving Sulfidibacter corallicola *and* Acanthopleuribacter pedis *genomes*

To improve the genome qualities of *S. corallicola*[159] and *A. pedis*[160], we produced fresh isolates of both strains. We cultivated the bacteria at 28 °C on 1/10 marine broth (MB) agar plates containing 3.74 g/L MB (BD Difco™ Marine Broth 2216), 30.6 g/L sea salts (Tropic Marin Pro-Reef), and 12 g/L agar (BD Difco™ Agar Noble). We inoculated 30 mL of liquid 1/10 MB medium containing 3.74 g/L MB (BD Difco™ Marine Broth 2216) and 30.6 g/L sea salts (Tropic Marin Pro-Reef) with a single colony and incubated the culture at 28 °C to an OD_{600} of 0.4. Genomic DNA was extracted using the Quick-DNA™ Miniprep Plus Kit (Zymo Research) and sent to Plasmidsaurus Inc. (UK) for hybrid long-read (Oxford Nanopore Technology) and short-read (Illumina) sequencing. Resulting *S. corallicola* and *A. pedis* genome assemblies and annotations—generated with the Plasmidsaurus pipeline—were submitted to ENA (PRJEB81969).

Testing holobiont–S. corallicola *interactions*

To examine the effect of the coral host on the growth, transcription profile, and actual metabolite production of an acidobacterial strain, we used the only acidobacterial strain isolated from a reef-building coral (*Porites lutea*) to date, *S. corallicola*[159] (of which we could match the 16S rRNA gene at 100% identity to a representative in our data). We performed an experiment as follows: A pre-culture of *S. corallicola* was prepared by inoculating 30 mL of 1/10 MB medium from a cryostock. After incubation at 28 °C with shaking (180 rpm) in the dark to an OD_{600} of 0.4, *S. corallicola* cells were washed three times by centrifugation (3,500 × *g*, room temperature, 5 min), resuspended in artificial seawater (34 g/L sea salts; Tropic Marin Pro-Reef), and then adjusted to an OD_{600} of 0.5. Using filtered calcium-/magnesium-free seawater (400 mM NaCl, 10 mM KCl, 7 mM Na_2SO_4, 0.005 mM $NaHCO_3$) and an airbrush at 5 bar, we blasted the tissue off of domestic *Porites* (procured from Zoologischer Garten Basel) fragments. We homogenised the tissue slurry using a Polytron PT 1200 E with the aggregate PT-DA 07/2SYN-E082 at maximum speed for a minute without cooling. Next, we performed two freeze–thaw cycles (-80 °C for 30 min and 35 °C until thawed completely) to disrupt the host cells. The resulting tissue slurry was added to dissolved organic matter exuded by *Porites* into autoclaved artificial seawater during 48 h to increase the volume and filtered three times. We prepared the culture medium as 1/10 full MB in 9/10 dissolved organic matter derived from *Porites* tissue extracts (or 9/10 artificial seawater for the control without coral organic matter). We again filtered both media (with and without coral additives) three times, kept an aliquot without bacteria as control (for growth curves and metabolite analysis), and spiked the remainder (980 mL) with 1 mL of the *S. corallicola* culture and divided the culture into three 1 L Erlenmeyer flasks per treatment. The flasks were incubated at 28 °C and gently shaken at 150 rpm in the dark. For enumerating the microbial cells, we stained bacteria with SYBR-Green I (final concentration 1:20,000) and incubated them in the dark and at room temperature for 20 min. For each sample, we recorded forward scatter (FSC) and green fluorescence (488 nm, SYBR) to identify bacterial cells. Flow rates did not exceed 30 µL/min to limit the abortion rate of sample during a 30 s sample acquisition time. Data were analysed with CytExpert (v2.5).

To measure the transcription profile, we sampled 50 mL of culture six days after inoculation (stationary phase; Extended Data Fig. 4d). After centrifugation (3,500 × *g*, room temperature, 5 min), we removed the supernatant by vacuum suction and immediately resuspended the

pellets in 450 µL of RLT lysis buffer (QIAGEN) with 1% □-mercaptoethanol (Sigma-Aldrich). Resuspended cells were transferred into 1.5 mL screw-capped tubes pre-filled with 600 mg of 100 µm Zirconium beads (OPS Diagnostics), plunged into liquid nitrogen, and stored at -80 °C for two weeks. RNA was extracted using the RNeasy Mini Kit (QIAGEN) and a QIAvac 24 Plus manifold (QIAGEN) following the QIAGEN RNeasy Mini Handbook (06/2023) and the protocols "Purification of Total RNA from Plant Cells and Tissues and Filamentous Fungi" and "Optional On-Column DNase Digestion with the RNAse-Free DNase Set". After thawing, the cells were subjected to bead beating (30 Hz, 5 min) with a Mixer Mill M400 (Retsch). Lysates were spun down and passed through a QIAshredder column (QIAGEN) by centrifugation (11,000 × *g*, 1 min) to remove beads. Cleared lysates (350 µL) were mixed with 350 µL ethanol (99.8%; Merck), passed through an RNeasy spin column followed by DNase-treatment (QIAGEN RNase-free DNase set) and washing. RNA extracts were eluted with two times 30 µL UltraPure DNase/RNase-Free Distilled Water (Invitrogen) and their integrity confirmed by TapeStation.

We sent the RNA extracts on dry ice to BMK (Biomarker Technologies GmbH, Germany) for library preparation and RNA sequencing. Briefly, ribosomal RNA was depleted with the RiboCop rRNA Depletion Kit (Lexogen) and the library constructed with the ALFA-SEQ Directional RNA Lib Prep Kit (Findrop) and sequenced on a NovaSeq 6000 platform (Illumina) to produce 3 Gbp of ~150 bp paired-end reads per sample. Raw RNA sequencing reads were cleaned by removing adaptor sequences, low-quality-end trimming, and removal of low-quality reads using BBTools (v38.18, Bushnell, B., BBMap, available from: https://sourceforge.net/projects/bbmap/). The exact commands used for quality control can be found on the Methods in Microbiomics webpage (Sintsova, A., Data Preprocessing — Methods in Microbiomics v0.0.1 documentation, available from: https://methods-in-microbiomics.readthedocs.io/en/latest/preprocessing/preprocessing.html). The quality-controlled reads were aligned against the *S. corallicola* genome assembly (ERZ25037212) using BWA aligner (v0.7.17)[109]. Transcript abundances were quantified using HTseq count (v2.0.2)[161]. This resulted in an average of 5.7M gene feature counts per sample (+/- 0.4M counts, $n = 6$). Differential gene expression analysis was performed using Bioconductor R package DESeq2 (v1.37.4)[162], which identified 291 genes that were differentially expressed when comparing cultures with and without coral additives (adjusted p-value < 0.05, absolute $\log_2$-fold change > 0.585). BGCs were annotated in the genome of *S. corallicola* (ERZ25037212) using antiSMASH (v7.1.0)[163], resulting in 44 predicted BGC regions.

Before chemically extracting the samples, we separated cells and supernatants by centrifugation (7,000 × *g*, 4 °C, 30 min). The supernatants were extracted with 3 × 300 mL of ethyl acetate and the combined organic phases were evaporated to dryness (yielding 15 mg and 6 mg for *S. corallicola* with and without coral additives, respectively). We also extracted 50 mL of the controls (no *S. corallicola*) with and without coral additives with 3 × 20 mL ethyl acetate, evaporating the organic phases to dryness (yielding 1 mg and 1 mg for medium with and without coral additives, respectively). The pellets were dissolved in 15 mL methanol and incubated with a magnetic stirrer for 1 h at 4 °C and 900 rpm. The pellets were then transferred to an ultrasonic water bath for 30 min and afterwards filtered and dried (yielding 67 mg and 23 mg for *S. corallicola* with and without additives, respectively). The supernatant, control, and pellet extracts were dissolved in methanol to obtain final

concentrations of 1.5 mg/mL, 1.5 mg/mL, and 6 mg/mL, respectively. Samples were analysed using high-performance liquid chromatography (HPLC; Dionex Ultimate 3000) coupled to heated electrospray ionisation high-resolution tandem mass spectrometry (HESI-HRMS/MS; Thermo Scientific Q Exactive) with the following gradient method: 99:1 A/B from 0–3 min, ramped to 5:95 A/B from 3–19 min, maintained at 5:95 A/B from 19–23 min, diminished to 99:1 A/B from 23–23.1 min, and maintained at 99:1 A/B from 23.1–25 min (solvent A: water + 0.1% formic acid; solvent B: acetonitrile + 0.1% formic acid; column: Kinetex™ 2.6 µm XB-C18 100 Å (150 × 4.6 mm); flow rate: 1 mL/min). Columns were maintained at 27 °C. The mass spectrometer was operated in positive ionisation mode at a scan range of 200–2000 *m/z* and a resolution of 17,500 (spray voltage: 3.5 kV; capillary temperature: 300 °C). Data were analysed using Xcalibur 4.1 (Thermo Fisher) and GNPS[164] to construct a molecular network.

We found that upon exposure to dissolved organic matter derived from *Porites* tissue extracts, *S. corallicola* transcribed more BGCs (Extended Data Fig. 4a) and produced a set of specific metabolites (Extended Data Fig. 4bc) while its growth was unaffected (Extended Data Fig. 4d). Whether similar BGC transcription and expression changes occur upon exposure to organic matter from other sources is currently unknown.

The RNAseq raw data were uploaded as project PRJEB81969. All files used for analysing holobiont–*S. corallicola* interactions were deposited on Zenodo (https://zenodo.org/doi/10.5281/zenodo.14050210)[146].

Functional characterisation of the *aci* cluster

In silico *analysis*

The *aci* BGC (TARA_SAMEA6023455_MAG_00000007-scaffold_11-biosynth_1), annotated by antiSMASH[162] and curated by sequence similarity searches[163], encodes two lanthipeptide precursors and a type III lanthionine synthetase homologue. AntiSMASH annotated this cluster as encoding a type IV lanthipeptide but closer examination revealed the lack of conserved zinc-binding motifs in AciKC (the lanthionine synthetase homologue), suggesting a type III lanthipeptide-encoding BGC. Additionally, genes encoding transcriptional regulators and ABC transporter related proteins are encoded in the BGC.

The proteins annotated as precursor proteins contain cysteine- and serine-rich putative core regions followed by unusual glycine-rich C-terminal regions, which, to our knowledge, are not yet described in type III lanthipeptides. The two precursors showed no homologues with significant similarity in the NCBI non-redundant protein sequence database.

Functional characterisation of AciKC

Functional characterisation was carried out by heterologous expression of genes encoding an N-terminal fusion of His_6-bdSUMO with either AciA1 or AciA2 by expression from a pACYC plasmid in *E. coli* BL21(DE3). In addition to the precursor genes, plasmid pET-24(+)-acikc was introduced and simultaneously expressed. Protein production and subsequent purification by nickel-nitrilotriacetic acid (Ni-NTA) and proteolysis by endoprotease GluC followed by liquid-chromatography-mass spectrometry (LC-MS)

revealed up to seven dehydrations (-126 Da) installed by AciKC on both precursors (Extended Data Fig. 6). Fragmentation ions of the proteolytically digested peptides suggested dehydration in the serine-rich putative core region (Extended Data Fig. 7), and no dehydrations in the C-terminal glycine-rich region (containing one to two serines theoretically available for dehydration in this region). The lack of b- and y-ions in the MS/MS fragmentation pattern in the putative core region suggested (multiple) macrocyclisation events through (methyl-)lanthionine and/or labionin formation.

Functional characterisation of the *tha* cluster

In silico *analysis*

The *tha* BGC (TARA_SAMEA6034818_MAG_00000048-scaffold_6), annotated by antiSMASH[165] and curated by sequence similarity searches[166], encodes a lanthipeptide precursor (*thaA*) that shares sequence similarity with mersacidin/lichenicidin family type 2 lantibiotics. Two small open reading frames on the boundaries of the cluster are annotated as putative lanthipeptides but share no sequence similarity to known proteins and were disregarded. Additionally, a gene encoding a class-II lanthionine synthetase homologue (*thaM)* is present as well as genes encoding transcriptional regulators and ABC-transporter related proteins and a C39 peptidase. Finally, a gene encoding a homologue of a GMC-family oxidoreductase (*thaO*) is located downstream of the lanthionine synthetase gene, suggesting that the enzyme could be part of the BGC. Multimeric AlphaFold2 predictions of the putative precursor with the enzymes suggested possible interactions with the core sequence predicted to bind near the active site (Extended Data Fig. 7). Genome neighbourhood diagrams[167,168] showed no clear evolutionary conserved cluster architectures when queried with the protein sequences for ThaA and ThaO.

Functional characterisation of ThaM

We heterologously expressed genes encoding an N-terminal fusion of His_6-bdSUMO with ThaA by expression from a pACYC plasmid in *E. coli* BL21(DE3). In addition to the precursor gene, plasmids pET-24(+)-tham, pET-24(+)-thao, or pET-24(+)-tham-thao were supplied and simultaneously expressed. Protein production and subsequent purification by Ni-NTA and proteolysis by trypsin followed by LC-MS revealed two dehydrations (-36 Da) installed by ThaM and a further mass loss of 20 Da when ThaM and ThaO were both produced (Extended Data Fig. 7). Expression of ThaO alone with ThaA did not lead to any apparent mass loss. Iodoacetamide derivatisation suggested formation of two lanthionine bridges in the twice dehydrated compound as only three (out of five) were modified by the reagent (Extended Data Fig. 6). The lack of fragmentation ions of Ser2 in the C-terminal region supported this observation (Extended Data Fig. 7). To investigate the ring topology, we produced mutants with each serine, threonine, and cysteine residue in the core region independently mutated to alanines. Out of the three amino acids suitable for dehydration, two were dehydrated. Tha-T6A was only dehydrated once, suggesting Thr6 to be dehydrated (Extended Data Fig. 7). While both Tha-S2A and Tha-T12A were dehydrated twice, Tha-S2A was only dehydrated once in co-production with ThaO and then further modified by ThaO, suggesting that while Ser2 is usually dehydrated, Thr12 can be dehydrated by ThaM in the absence of Ser2. This is supported by the lack of fragmentation

ions from Ser2 to the C-terminus in the native ThaA precursor (Extended Data Fig. 7). Mutants Tha-S2A and Tha-C16A produced similar fragmentation ions, as did mutants Tha-T6A and Tha-C13A, suggesting that Ser2 formed a lanthionine with Cys16, and Thr6 formed a lanthionine with Cys13 (Extended Data Fig. 7). At the same time, these results suggested only partial lanthionine formation of both macrocycles in these mutants. The absolute configuration was determined by Marfey's analysis and revealed D,L-lanthionine and D,L-methyllanthione formation (Extended Data Fig. 6).

Functional characterisation of ThaO

The exact mass loss catalysed by ThaO corresponded to 20.0272 Da in TB. We conducted co-expression of genes for the precursor and the enzymes in ^{13}C- and ^{15}N-labelled medium and observed the same mass loss, 20.0312 Da in ISOGRO-^{15}N and 20.0270 Da in ISOGRO-^{13}C (Extended Data Fig. 7). This excluded carbon and nitrogen loss and led us to believe a loss of four protons and one oxygen atom as the likely source of the 20 Da mass loss. Tandem MS fragmentation suggested that the mass loss can be localised to the glycine–cysteine residues adjacent to the dehydrated serine residue, with the presence of unmodified y_{15} and modified y_{17}–y_{21} ions in the AspN digest, and y_{15} and y_{18}, y_{19}, and y_{21} ions in the trypsin digest, respectively (Extended Data Fig. 7). Cysteine derivatisation by iodoacetamide and Raney nickel independently supported that only two cysteines are available compared to three cysteines in the dehydrated compound (Extended Data Fig. 6). Point mutation of Cys1 to Ala abolished production of the further modified compound and was only dehydrated twice, further supporting that Cys1 (together with the adjacent Gly-1) is modified by ThaO (Extended Data Fig. 7). To characterise the modification, we performed NMR experiments that revealed a peak in the ^{1}H-NMR at 8.20 ppm, coupled to a ^{13}C in the HSQC at 124.27 ppm, and two ^{13}C at 148.63 and 170.33 ppm in the HMBC spectra (Extended Data Fig. 8). These peaks are characteristic for thiazole formation and are in agreement with previously reported thiazole-containing peptides[169,170]. Taken together, these findings support that ThaO catalyses unusual thiazole formation in ThaA in the presence of ThaM. FAD-dependent oxidation of cysteine to a thioaldehyde, followed by thioenolisation, nucleophilic ring closure onto the amide carbonyl, and subsequent elimination of H_2O is in agreement with FAD-dependent cysteine oxidation observed in the initial steps of aminovinyl-cysteine-containing peptide biosynthesis[171,172,173]. Widespread thiazole and (methyl-)oxazole biosynthesis in RiPPs is typically catalysed by a pair of enzymes, a YcaO-domain containing enzyme catalysing ring formation and a dehydrogenase catalysing ring oxidation[49,174].

Functional characterisation of the homologous *the* cluster

In silico *analysis*

To further explore the family of RiPP-modifying GMC-oxidoreductases, we searched for homologues of ThaO through blastp against the NCBI nr database. We manually curated hit sequences and selected BGCs encoding for precursor sequences with additional amino acids between GGC motifs and the first serine or threonine in the putative core peptide for closer analysis. We reasoned that this strategy might yield GMC-oxidoreductase homologues that are more independent of lanthionine synthetase encoded in the same

BGC. The selected *the* BGC (MAG, GenBank accession: GCA_035278895.1), annotated by antiSMASH[165] and curated by sequence similarity searches[166], encodes a lanthipeptide precursor (*theA*) that shares sequence similarity with mersacidin/lichenicidin family type 2 lantibiotic precursors. The cluster further encodes a GMC-family oxidoreductase (*theO*) and a class-II lanthionine synthetase homologue (*theM)* as well as a gluconate 2-dehydrogenase subunit 3-like protein (*theB*) and a GCN5-related *N*-acetyltransferase homologue (*theN*).

Functional characterisation of TheO

Upon co-production of an N-terminal fusion of His_6-bdSUMO and TheA with TheO, we observed a 20.0247 Da mass loss, suggesting similar thiazole formation as observed for ThaA co-produced with ThaO in the *tha* cluster (Extended Data Fig. 9). Tandem MS fragmentation and mutations of glycine residues near the putatively modified cysteine further supported thiazole formation as the source of the 20 Da loss. In a series of C-terminal truncations of the TheA core, we observed wildtype-like efficiency for $TheA_{20}$, $TheA_{14}$, and $TheA_8$, which consist of 20, 14, or 8 amino acid long core sequences, respectively (Extended Data Fig. 9). Further truncations were not modified by TheO. For production of a cargo-fused precursor, we fused the Protegrin-1 sequence to the C-terminus of the $TheA_8$ truncation to minimise the distance between the thiazole-forming site and the protein cargo. Upon co-production of this fusion protein, we observed the same mass loss of 20.0228 Da in the tryptic fragment, suggesting thiazole formation N-terminal of the Protegrin-1 sequence (Extended Data Fig. 9).

Binomial naming of new acidobacterial lineages

For the three acidobacterial species represented by long-read MAGs, we propose the following names based on the International Code of Nomenclature of Prokaryotes (ICNP) and SeqCode[175] recommendations:

MAG48; TARA_SAMEA6034818_MAG_10000048; GCA_977880245 (one contig)

'*Candidatus* Moanaiibacterium' (Mo.a.na.i.i.bac.te'ri.um. N.L. neut. n. Moanaiibacterium, a composite of (Hine)Moana—Latinised as Moanaia, gen. Moanaiae, the Polynesian goddess of the sea, and bacterium; referring to the oceanic origin of the bacterial taxon);

'*Candidatus* Moanaiibacterium opulentum' (o.pu.len'tum. N.L. neut. adj. opulentum, rich, abundant, referring to the numerous biosynthetic gene clusters identified as well as the large genome size) is proposed as the type species of the genus *Ca*. Moanaiibacterium, which is represented by a long-read, chromosome-level metagenome-assembled genome as type material.

MAG13; TARA_SAMEA6035815_MAG_10000013; GCA_977880235 (two contigs)

'*Candidatus* Tangaroaiibacterium' (Tan.ga.ro.i.bac.te'ri.um. N.L. neut. n. Tangaroaiibacterium, a composite of Tangaroa—Latinised as Tangaroaius, gen. Tangaroaii, the Polynesian god of the sea, and bacterium; referring to the oceanic origin of the bacterial taxon);

‘*Candidatus* Tangaroaiibacterium tarapacificum’ (ta.ra.pa.ci'fi.cum. N.L. neut. adj. tarapacificum, referring to the *Tara* Pacific expedition from which the genome was recovered) is proposed as the type species of the genus *Ca*. Tangaroaiibacterium, which is represented by a long-read, chromosome-level metagenome-assembled genome as type material.

MAG20; TARA_SAMEA6034818_MAG_10000020; GCA_977880255 (seven contigs)

‘*Candidatus* Taniwhaiibacterium’ (Ta.niw.ha.i.i.bac.te'ri.um. N.L. neut. n. Taniwhaiibacterium, a composite of Taniwha—Latinised as Taniwhaius, gen. Taniwhaii, guardians of the sea, reefs, and other water bodies in Polynesian mythology, and bacterium; referring to the oceanic origin of the bacterial taxon);

‘*Candidatus* Taniwhaiibacterium abditum’ (ab.di'tum. N.L. neut. adj. abditum, hidden, referring to the more sporadic distribution of the taxon) is proposed as the type species of the genus *Ca*. Taniwhaiibacterium, which is represented by a long-read, chromosome-level metagenome-assembled genome as type material.

Proposed higher level taxonomy

On the basis of these clades, we further propose ‘*Candidatus* Moanaiibacteriaceae’ (fam. nov.), ‘*Candidatus* Moanaiibacteriaceae’ (fam. nov.), ‘*Candidatus* Tangaroaiibacteriales’ (ord. nov.), and ‘*Candidatus* Tangaroaiibacteriia’ (class nov.).

Research (UNCLOS) permits

Sampling permit for PANAMA under the reference 'SE/AP-18-16' delivered by the Direccion de Areas Protegidas y Vida Silvestre - LIC. Samuel Valdez Diaz Director - Ministerio de Ambiente – Republica de Panama on the 13/06/2016; Sampling permit for PANAMA under the reference '2016-0701-2019-2' delivered by the Smithsonian Tropical Research Institute Instituto Smithsonian de Investigaciones Tropicales - STRI Animal Care and Use Committee (ACUC) on the 28/06/2016; Sampling permit for PANAMA under the reference '2016-0701-2019-2-A1' delivered by the Smithsonian Tropical Research Institute Instituto Smithsonian de Investigaciones Tropicales - STRI Animal Care and Use Committee (ACUC) on the 21/06/2018; Sampling permit for COLOMBIA under the reference 'N°009' delivered by the MINISTERIO DE AMBIENTE Y DESARROLLO SOSTENIBLEPARQUES NACIONALES NATURALES DE COLOMBIA on the 04/03/2016; Sampling permit for CHILE under the reference '13270/24/457/Vrs' delivered by the Servicio Hidrografico y Oceanografico de la Armada de Chile (SHOA) – Patricio Carrasco Hellwig Contraalmirante Director on the 29/08/2016; Sampling permit for UNITED-KINGDOM (PITCAIRN ISLANDS) under the reference 'N/A' delivered by the Government of Pitcairn islands /Environmental, Conservation & Natural Resources Division Manager // Christian Michele on the 25/02/2016; Sampling permit for COOK under the reference '11-16' delivered by the Foundation for National Research – Cook Island Research Committee – Office of the Prime Minister – Elizabeth Wright-Koteka (Chairperson) on the 12/09/2016; Sampling permit for NIUE under the reference '34/16' delivered by the Government of Niue – Office for External Affairs on the 17/11/2016; Sampling permit for SAMOA under the reference 'Memorandum of Agreement' delivered by the THE GOVERNMENT OF THE INDEPENDENT STATE OF SAMOA acting

by and through the Ministry of Natural Resources and Environment on the 29/11/2016; Sampling permit for WALLIS AND FUTUNA under the reference 'Arrêté n°2016-527' delivered by the Le Préfet, Administrateur supérieur des îles Wallis et Futuna on the 24/11/2016; Sampling permit for TUVALU under the reference 'MFAT : 449/16' delivered by the Government of Tuvalu – Ministry of Foreign Affairs on the 19/12/2016; Sampling permit for KIRIBATI under the reference '015/16' delivered by the Environment and Conservation Division – Republic of Kiribati on the 24/11/2016; Sampling permit for MICRONESIA under the reference 'Letter' delivered by the Deputy Assistant Secretary – Marine Resources Unit – Department of Resources and Development – Federated States of Micronesia on the 05/04/2017; Sampling permit for GUAM under the reference 'U2021-023' delivered by the Marine Scientific Research Coordinator Office of Ocean and Polar Affairs – United States Department of State Bureau of Oceans and International Environmental and Scientific Affairs on the 27/10/2021; Sampling permit for AMERICAN SAMOA under the reference 'U2021-022' delivered by the Marine Scientific Research Coordinator Office of Ocean and Polar Affairs – United States Department of State Bureau of Oceans and International Environmental and Scientific Affairs on the 27/10/2021; Sampling permit for JAPAN (Tokyo Prefecture; Ogasawara Island) under the reference '28-50' delivered by the Prefecture of Tokyo on the 01/23/2017; Sampling permit for JAPAN (Okinawa Prefecture; Sesoko Island) under the reference '28-74' delivered by the Prefecture of Okinawa on the 04/14/2017; Sampling permit for JAPAN (Japanese EEZ) under the reference 'N/A' delivered by the Ministry of Agriculture, Forestry and Fisheries on the 01/10/2017; Sampling permit for FIJI under the reference '456/2017' delivered by the Ministry of Foreign Affairs – Republic of Fiji on the 11/06/2017; Sampling permit for AUSTRALIA under the reference 'G17/39873.1' delivered by the Great Barrier Reef Marine Park Authority and Department of Foreign Affairs and Trade on the 30/08/2017; Sampling permit for NEW-CALEDONIA (SOUTH PROVINCIA) under the reference 'Arrêté n°2720-2017/ARR/DENV modifiant l'arrêté 1515-2017/ARR/DENV du 04 août 2017' delivered by the Président de l'Assemblée de la Province Sud de la Nouvelle-Calédonie on the 06/09/2017; Sampling permit for NEW-CALEDONIA (CHESTERFIELD) under the reference 'Arrêté n°2017-2069/GNC' delivered by the Haut-Commissariat de la République en Nouvelle-Calédonie – Gouvernement de Nouvelle-Calédonie – République Française on the 29/08/2017; Sampling permit for SOLOMON ISLANDS under the reference 'Form 01' delivered by the Solomon Islands Maritime Safety Administration on the 20/09/2017; Sampling permit for PAPUA NEW-GUINEA under the reference '907/2017 (diplomatic clearance n°0232)' delivered by the Department of Foreign Affairs and Trade of the Independent State of Papua New Guinea on the 27/10/2017; Sampling permit for PALAU under the reference 'RE-18-04' delivered by the Ministry of Natural Resources, Environment and Tourism – Republic of Palau on the 21/12/2017; Sampling permit for CHINA (HONG-KONG) under the reference 'CMO-N00811' delivered by the Marine Department, Hong-Kong, China on the 15/03/2018; Sampling permit for TAIWAN (Pingtung county) under the reference '10707821600' delivered by the Pingtung Agri-Fish; National Taiwan Ocean University on the 06/04/2018; Sampling permit for TAIWAN (Taitung county) under the reference '1070033041' delivered by the Taitung Agri-Fish; National Taiwan Ocean University on the 12/02/2018; Sampling permit for USA (HAWAII) under the reference 'U2018-010' delivered by the United States Department of State Bureau of Oceans and International Environmental and Scientific Affairs on the 06/06/2018; Sampling permit for MEXICO under the reference 'PPF/DGOPA-291/17' delivered by the Secrataria de Agricultura, Ganaderia, Desarrollo rural, pesca y alimentacion

– Comision Nacional de Acuacultura y Pesca – Direccion General de Ordenamiento Pesquero y Acuicola – Estados Unidos Mexicanos on the 28/08/2018; Sampling permit for CLIPPERTON under the reference 'HC/1195/CAB' delivered by the Haut-Commissariat de la République Polynésie Française on the 13/06/2018; Sampling permit for COSTA RICA under the reference M-C-SINAC-PNI-SE-002-2022 delivered by the Sistema Nacional de Áreas de Conservación (SINAC) on the 29/09/2022; Sampling permit for USA (MAINLAND) under the reference 'U2018-010' delivered by the United States Department of State Bureau of Oceans and International Environmental and Scientific Affairs on the 06/06/2018; Sampling permit for CANADA under the reference 'Letter of regularization' delivered by the Sécurité et relations de défense (IGR)/Affaires mondiales Canada on the 05/01/2022; Sampling permit for NEW-ZEALAND under the reference 'Letter of regularization' delivered by the Ministry of Foreign Affairs and Trade on the 05/10/2021; Sampling permit for IRELAND under the reference '572/22' delivered by the Department of Foreign Affairs on the 09/06/2022.

CITES permits

CITES export permit for PANAMA (I01) under the reference 'SEX/A-72-16' delivered by the Autoridad Nacional del Ambiente (ANAM) de la República de Panamá – Autoridad Administrativa CITES on the 28/07/2016; CITES final import permit under the reference 'FR1609100066-I' delivered the 04/08/2016 by the DRIEE ILE-DE-FRANCE; CITES export permit for PANAMA (I02) under the reference 'SEX/A-72-16' delivered by the Autoridad Nacional del Ambiente (ANAM) de la República de Panamá – Autoridad Administrativa CITES on the 28/07/2016; CITES final import permit under the reference 'FR1609100066-I' delivered the 04/08/2016 by the DRIEE ILE-DE-FRANCE; CITES export permit for PANAMA (I31) under the reference 'SEX/APO-1-2018' delivered by the Ministerio de Ambiente on the 30/08/2018; CITES final import permit under the reference 'FR1807523129-I' delivered the 19/10/2018 by the DRIEE ILE-DE-FRANCE; CITES export permit for PANAMA (I32) under the reference 'SEX/APO-1-2018' delivered by the Ministerio de Ambiente on the 30/08/2018; CITES final import permit under the reference 'FR1807523129-I' delivered the 19/10/2018 by the DRIEE ILE-DE-FRANCE; CITES export permit for COLOMBIA (I03) under the reference '41499' delivered by the Ministerio de Ambiente y Desarrollo Sostenible de la República de Colombia on the 13/02/2017; CITES final import permit under the reference 'FR1707506158-I' delivered the 17/03/2017 by the DRIEE ILE-DE-FRANCE; CITES export permit for CHILE (I04) under the reference '16CL000007WS' delivered by the Servicio Nacional de Pesca y Acuicultura on the 02/09/2016; CITES final import permit under the reference 'FR1607525599-I' delivered the 03/11/2016 by the DRIEE ILE-DE-FRANCE; CITES export permit for UNITED-KINGDOM (PITCAIRN ISLANDS; I05) under the reference 'FR1698700198-E' delivered by the Haut-Commissariat de la République en Polynésie Française on the 03/11/2016; CITES final import permit under the reference 'FR1607525646-I' delivered the 04/11/2016 by the DRIEE ILE-DE-FRANCE; CITES export permit for FRENCH POLYNESIA (GAMBIER – TUAMOTU; I06) under the reference 'FR1698700198-E' delivered by the Haut-Commissariat de la République en Polynésie Française on the 03/11/2016; CITES final import permit under the reference 'FR1607525646-I' delivered the 04/11/2016 by the DRIEE ILE-DE-FRANCE; CITES export permit for MOOREA (I07) under the reference 'FR1698700218-E' delivered by the Haut-Commissariat de la République en Polynésie Française on the 21/11/2016; CITES final

import permit under the reference 'FR1707503441-I ' delivered the 07/02/2017 by the DRIEE ILE-DE-FRANCE; CITES export permit for COOK (I08) under the reference 'CK/2016 – 14278' delivered by the Tu'anga Taporoporo national environment service of the Cook Islands on the 17/11/2016; CITES final import permit under the reference 'FR1707503442-I ' delivered the 07/02/2017 by the DRIEE ILE-DE-FRANCE; CITES export permit for NIUE (I09) under the reference 'N/A' delivered by the N/A on the N/A; CITES final import permit under the reference 'FR1707511900-I' delivered the 11/06/2017 by the DRIEE ILE-DE-FRANCE; CITES export permit for SAMOA (I10) under the reference 'SAMC16012' delivered by the Ministry of Natural Resources and Environment (MNRE) of the Government of Samoa on the 29/11/2016; CITES final import permit under the reference 'FR1707503440-I' delivered the 07/02/2017 by the DRIEE ILE-DE-FRANCE; CITES export permit for WALLIS AND FUTUNA (I11) under the reference 'WF/C/16/01' delivered by the Préfet – Admistrateur Supérieur – Chef du territoire des Îles Wallis et Futuna on the 25/12/2016; CITES final import permit under the reference 'FR1707503441-I ' delivered the 07/02/2017 by the DRIEE ILE-DE-FRANCE; CITES export permit for TUVALU (I12) under the reference '1204 (Quarantine document)' delivered by the Plant Protection and Quarantine Services - Ministry of Natural Resources - TUVALU GOVERNMENT on the 03/01/2017; CITES final import permit under the reference 'FR1707511900-I' delivered the 11/06/2017 by the DRIEE ILE-DE-FRANCE; CITES export permit for KIRIBATI (I13) under the reference '015/16 (UNCLOS permit)' delivered by the Fisheries Division, Ministry of Fisheries & Marine Resources Development -- GOVERNMENT OF KIRIBATI on the 12/01/2017; CITES final import permit under the reference 'FR1707511900-I' delivered the 11/06/2017 by the DRIEE ILE-DE-FRANCE; CITES export permit for MICRONESIA (CHUUK; I14) under the reference 'CFM17-01-01' delivered by the Department of Resources and Development – Division of Resource Management and Development – Office of Marine Resources on the 19/01/2017; CITES final import permit under the reference 'FR1707511900-I' delivered the 11/06/2017 by the DRIEE ILE-DE-FRANCE; CITES export permit for GUAM (I15) under the reference '17US18844C/9' delivered by the U.S. Fish and Wildlife service – Division of management authority – Branch of permits on the 02/03/2017; CITES final import permit under the reference 'FR1707503440-I' delivered the 07/02/2017 by the DRIEE ILE-DE-FRANCE; CITES export permit for JAPAN (OGASAWARA; I16) under the reference '17JP001279/TE' delivered by the Trade and Economic Cooperation Bureau – Ministry of Economy, Trade and Industry (METI) on the 16/05/2017; CITES final import permit under the reference 'FR1707511899-I' delivered the 06/06/2017 by the DRIEE ILE-DE-FRANCE; CITES export permit for JAPAN (SESOKO; I17) under the reference '17JP001280/TE' delivered by the Trade and Economic Cooperation Bureau – Ministry of Economy, Trade and Industry (METI) on the 16/05/2017; CITES final import permit under the reference ' FR1707511898-I ' delivered the 06/06/2017 by the DRIEE ILE-DE-FRANCE; CITES export permit for FIJI (I18) under the reference 'FJ/EXP-03055' delivered by the Fisheries Department - Government of Fiji on the 08/06/2017; CITES final import permit under the reference 'FR1707521006-I' delivered the 27/09/2017 by the DRIEE ILE-DE-FRANCE; CITES export permit for AUSTRALIA (I19) under the reference 'PWS2017-AU-001613 ' delivered by the Department of the Environment and Energy of the Australian Government on the 22/08/2017; CITES final import permit under the reference 'FR1707521097-I' delivered the 29/09/2017 by the DRIEE ILE-DE-FRANCE; CITES export permit for NEW-CALEDONIA (SOUTH PROVINCIA; I21) under the reference 'FR1798800075-E' delivered by the Haut-Commissariat de la République en

Nouvelle-Calédonie / DAFE on the 22/09/2017; CITES final import permit under the reference 'FR1707521096-I' delivered the 29/09/2017 by the DRIEE ILE-DE-FRANCE; CITES export permit for NEW-CALEDONIA (CHESTERFIELD; I20) under the reference 'FR1798800075-E' delivered by the Haut-Commissariat de la République en Nouvelle-Calédonie / DAFE on the 22/09/2017; CITES final import permit under the reference 'FR1707521096-I' delivered the 29/09/2017 by the DRIEE ILE-DE-FRANCE; CITES export permit for SOLOMON ISLANDS (I22) under the reference 'EX2017/188' delivered by the Ministry of Environment, Climate Change, Disaster Management and Met on the 19/10/2017; CITES final import permit under the reference 'FR1807501178-I' delivered the 15/01/2018 by the DRIEE ILE-DE-FRANCE; CITES export permit for PAPUA NEW-GUINEA (I23) under the reference '18004' delivered by the Conservation and Environment Protection Authority (CEPA) on the 06/12/2017; CITES final import permit under the reference 'FR1807508641-I' delivered the 24/04/2018 by the DRIEE ILE-DE-FRANCE; CITES export permit for PAPUA NEW-GUINEA (I24) under the reference '18004' delivered by the Conservation and Environment Protection Authority (CEPA) on the 06/12/2017; CITES final import permit under the reference 'FR1807508641-I' delivered the 24/04/2018 by the DRIEE ILE-DE-FRANCE; CITES export permit for PALAU (transect; I25) under the reference 'PW18-004' delivered by the Office of the Minister – Ministry of Natural Resources, Environment and Tourism on the 10/01/2018; CITES final import permit under the reference 'FR1807501177-I' delivered the 16/01/2018 by the DRIEE ILE-DE-FRANCE; CITES export permit for PALAU (leg; I25) under the reference 'PW18-009' delivered by the Office of the Minister – Ministry of Natural Resources, Environment and Tourism on the 01/10/2018; CITES final import permit under the reference 'FR1807512823-I' delivered the 13/06/2018 by the DRIEE ILE-DE-FRANCE; CITES export permit for CHINA (HONG-KONG; I26) under the reference 'APO/EL 3/18' delivered by the Agriculture, Fisheries and Conservation Department of Hong-Kong Special Administrative Region on the 16/04/2018; CITES final import permit under the reference 'FR1807508518-I' delivered the 23/04/2018 by the DRIEE ILE-DE-FRANCE; CITES export permit for TAIWAN (I27) under the reference 'FTS507W0147330' delivered by the Bureau of Foreign Trade – Ministry of Economic Affairs on the 21/06/2018; CITES final import permit under the reference 'FR1807515565-I' delivered the 18/07/2018 by the DRIEE ILE-DE-FRANCE; CITES export permit for USA (HAWAII; I28) under the reference '18US97917C/9' delivered by the U.S. Fish and Wildlife service – Division of management authority – Branch of permits on the 26/07/2018; CITES final import permit under the reference 'FR180751766-I' delivered the 09/08/2018 by the DRIEE ILE-DE-FRANCE.

Acknowledgment of local authorities

We thank all countries and their residents listed below, with special acknowledgement to the local institutions and supporters mentioned by name for their invaluable administrative and scientific support, which made this exceptional expedition possible:

Australia: the Department of Foreign Affairs and Trade with Madeleine Summers; the Great Barrier Reef Marine Park Authority with Director Kirstin Dobbs, Senior Project Officer Thea Waters, and Senior Investigator Matthew Slatcher; the Queensland Parks and Wildlife Service; the Department of the Environment and Energy, particularly the Wildlife Trade Office with Senior Wildlife Officer Ros Wilkins; Geoscience Australia; the Global Change

Institute of the University of Queensland with Professor and Director Ove Hoegh-Guldberg FAA; and all other local supporters.

China: the Director of Marine of the Marine Department of Hong Kong; the State Oceanic Administration of the International Cooperation Department with Xinwei Yu; the Swire Institute of Marine Science of the University of Hong Kong with Till Roethig, David Baker, Vriko Yu, and Ms Ho; the Coral Reef Research Center of China of the Guangxi University with Professor Yu Kefu and Chen Biao (Ph.D.); the Agriculture, Fisheries and Conservation Department of the Hong Kong Special Administrative Region with Azaria K.Y. Wong.

Chile: the Subsecretaria de pesca y acuicultura - Fomento y tourismo del Ministerio de Economia; the Direccion de Medio ambiente y asuntos Oceanicos - Ministerio de Relaciones Exteriores; la Armada de Chile - Servicio hidrografico y oceanografico; the Comité Nacional CITES with her Coordinator Céspedes Lagos Nancy; the Servicio nacional de Pesca y Acuicultura (SERNAPESCA) - Autoridad Administrativa CITES with Palma Antonio.

Colombia: the Office of the Prime Minister; the Ministry of Environment and National Natural Parks of Colombia with Guillermo Alberto Santos Ceballos and Edna Carolina Jarro Fajardo; the Fundación Malpelo y Otros Ecosistemas Marinos with their Executive Director and Founder Sandra Bessudo and Felipe Orlando Ladino; the Santuario de Fauna y Flora Malpelo; the Dirección General Marítima with Ivan Fernando Castro Mercado; the Pacific Territorial Directorate; the Biodiversity Information System of Colombia.

Costa Rica: the Ministerio de Ambiente y Energia, and in particular the Comisión Nacional para la Gestión de la Biodiversidad (CONAGEBIO) and its Oficina Técnica (OT), with M.Sc. Angela González Grau, M.Sc. José Hernández Ugalde, Licda. Grettel Céspedes Arias, M.Sc. Melania Muñoz García; the Sistema Nacional de Áreas de Conservación (SINAC), with Gustavo Induni Alfaro; Juan José Alvarado Barrientos from the Centro de Investigación en Ciencias del Mar y Limnología (CIMAR).

Fiji: the Government of the Republic of Fiji; the Ministry of Foreign Affairs; the Ministry of Fisheries and Forests and its Offshore Division with Unaisi Rabici; the Department of Environment with Krystelle Suliano; the School of Marine Studies of the University of the South Pacific with Amandine Marie (Ph.D.); the Alliance Française of Suva.

New Caledonia: the Government of New Caledonia; the Department of Regional Cooperation and External Relations with Chief François Bockel and Anne-Claire Goarant; the Department of Knowledge, Biodiversity and Territories with Chief Isabelle Jurquet; the Southern Province Administration with Melusine Lefebvre, Emmanuel Coutures, Sylvie Goyet, and Margot Mesnard; the Research Institute for Development with Francesca Benzoni; the SCA-LAIR project with Eric Le Plomb; the Natural Park of the Coral Sea Administration; the Maritime Affairs Directorate and the Fisheries and Marine Environment Service with Julie-Anne Kerandel; the Department of Agriculture, Forestry and the Environment of the State Department with Franck Connan and Karine Accou.

French Polynesia: the High Commissioner of the Republic of French Polynesia; the City of Papeete with its Mayor and Port Commander; the Centre de Recherches Insulaires et Observatoire de l’Environnement.

Clipperton: the High Commissioner of the Republic of French Polynesia, its Cabinet, and the Chief of Service Maxime Gutzwiller; the Regional Delegate of Research and Technology; the Hydrographic and Oceanographic Service of the Navy; the French Research Institute for the Exploitation of the Sea; the French Office for Biodiversity; Météo-France; the CEREMA.

Wallis and Futuna: the Prefecture of Wallis and Futuna with Directeur des Affaires Maritimes Viane Hoatau; the Environment Service with Chief Atoloto Malau and its successor Vanai Paino.

Japan: the Ministry of Economy, Trade and Industry and its CITES Management Authority with Mitsuha Aoyagi; the Shimoda Marine Research Center of the University of Tsukuba with Sylvain Agostini; the Uyeno Marine Service K.K. with Yoneyama Yusuke and Tsumura Masashi.

Kiribati: the Ministry of Foreign Affairs and Immigration with Assistant Protocol Officer Kaee Itaaka Mackenzie; the Ministry of Fisheries and Marine Resources Development with the Director of the Fisheries Division Tooreka Temari and T. Aram Karibanang; the Ministry of Environment, Lands and Agricultural Development and its Environment and Conservation Division with Victoria Hnanguie; Marine Conservation Officer Chimres Teresio.

Mexico: the Secretaría de Relaciones Exteriores and the Agencia Mexicana de Cooperación Internacional para el Desarrollo; the Centro Interdisciplinario de Ciencias Marinas del Instituto Politécnico Nacional; the Universidad Autonoma de Baja California Sur and the Director del Programa de Investigación para la Conservación de la Fauna Arrecifal Carlos A. Sanchez Ortiz (Ph.D.); the National Commission of Natural Protected Areas; the Directorate General of Fisheries and Aquaculture Management; the National Commission of Aquaculture and Fisheries; the Director General (Autoridad Expedidora) Victor Manuel Arriaga Horo of the Secrataría de Agricultura, Ganadería, Desarollo Rural, Pesca y Alimentación; the Centro Regional de Investigación Pesquera en La Paz; the Laboratorio de Necton y Ecología de Arrecifes of the Centro de Investigaciones Biológicas del Noroeste with David Arturo Paz Garcia.

Federated States of Micronesia: the Federated States of Micronesia; the Governor’s Cabinet; the Chuuk State Leadership; the Chuuk State Office of Public Affairs; the Division of Immigration and Labour; the Marine Conservation Officers, particularly Chimres Teresio; the Department of Resources and Development with Director Curtis Graham, Deputy Assistant Secretary of the Marine Resources Unit Valentin A. Martin, and Marine Conservation Management Specialist Dave Mathias.

Cook Islands: the Government of the Cook Islands; the Office of the Prime Minister with Tina Samson and Elizabeth Wright-Koteka from the Cook Islands Research Committee; the Ministry of Marine Resources with Director Dorothy Solomona; the National Environment Service with Director Joseph Brider, Elizabeth Munro, and Bobby Bishop; the City of Aitutaki, particularly its mayor; the Aitutaki Marine Research Station with its Station Manager Richard Story; the Cawthron Institute with Lesley Rhodes and Kirsty Smith.

Niue: the Government of Niue with Richard Hipa, James Tatafu, and Aldric Hipa; the Office of External Affairs of the Government of Niue with Director Emi Hipa; the Cabinet and

Parliamentary Services with Director Christine External; the Ministry of Infrastructure and its Department of Transport with Lynsey Talagi; the Ministry of Natural Resources with Director General Josie Tamate; the Department of Agriculture, Forestry and Fisheries at the Ministry of Natural Resources with Director Brendon Pasisi; the Department of Environment of the Ministry of Natural Resources and Director Sauni Tongatule; the Pacific Community with Deputy Director General Cameroun Diver and Coral Pasisi.

Palau: the President of the Republic of Palau, Tommy E. Remengesau, and his Office; the Chief of Staff of the Republic of Palau, Secilil Eldebechel; the Ministry of Natural Resources, Environment and Tourism with Minister F. Umiich Sengebau, Special Assistant King M. Sam, and the Office of the Minister; the Bureau of Marine Resource of the Republic of Palau with Coastal Fisheries Officer Jerome Oiterong; the Governor of the Koror State Government of the Republic of Palau with the Honorable Franco Gibbons; the Koror State Rangers with Chief Ranger Jennifer Olgeriil; the Palau International Coral Reef Center with its CEO Yimnang Golbuu (Ph.D.) and Scientist Geraldine Rengiil; the Palau National Marine Sanctuary with Executive Director Keobel V. Sakuma; Son Altesse Sérénissime le Prince Souverain de Monaco, Prince Albert II; the Centre Scientifique de Monaco.

Panama: the Ministry of Environment of Panama with Samuel Valdès Diaz, Lissette Trejos, and Patricia Hernandez; the Smithsonian Tropical Research Institute with Rachel Page, Zurenayka Alain, and Juan Mate; the Head of MPA at Coiba Park Didiel Nunez.

Papua New-Guinea: the Department of Foreign Affairs and Trade with Director Morea Veratau; the Marine Research Scientific Committee with Chairman Russell Clark Perembo (Ph.D.) of the Division of Earth Sciences; the National Research Institute with Commercial Services Manager Georgia Kaipu; the Conservation and Environment Protection Authority with Barnabas Wilmot; the University of Papua New-Guinea with Head of Biological Sciences Pr. Simon Saulei; the Wildlife Conservation Society with Country Director Ambroise Brenier; the Walindi Plantation Resort with Benjamin Cheyne.

Samoa Islands: the Government of the Independent State of Samoa; the Ministry of Foreign Affairs and Trade with CEO Peseta Noumea Simi, Acting CEO Leroy E. Hunkin-Mamae, and Sapeti Titiii; the Ministry of Natural Resources and Environment with Principal Marine Conservation Officer Maria R. Satoa Peni; the Ministry of Agriculture and Fisheries with Assistant CEO Magele Etuati Ropeti; the local population of Samoa.

Solomon Islands: the Ministry of Foreign Affairs and External Trade; the Ministry of Environment, Climate Change, Disaster Management, Meteorology, and its Environment and Conservation Division with Deputy Director Josef Hurutarau and Conservation Officer Judah Viravira; the Maritime Safety Administration; the Coastal Marine Management with Hughes Alec; the GS Agencies with Managing Director Gerald Stenzel.

Taiwan: the National Taiwan Ocean University with President Ching-Fong Chang (Ph.D.), Cheih Jhen Chen (Erin), Irisa, Vianney Denis, and the Assistant Professor at the Institute of Oceanography; the Alliance Française of Taipei with Director Benoit Guidée; the City of Keelung, particularly its Mayor.

Tuvalu: the High Commissioner of Tuvalu, H.E. Paulson Panapa; the Government of Tuvalu; the Ministry of Foreign Affairs, Trade, Tourism, Environment and Labour with Temate Melitiana; the Department of Foreign Affairs of the Ministry of Foreign Affairs, Trade, Tourism, Environment and Labour with Bilateral and Regional Affair Officer Falata Kilisi; the Senior Plant Protection & Quarantine with Matio Lonalona and Pauke Pasivao.

Pitcairn Islands: the Government of Pitcairn Islands with Melva Evans; the Office of the Mayor and Council for Pitcairn, Henderson, Ducie and Oeno Islands with Shawn Christian; the Environment, Conservation and Natural Resource Division with Manager Michele Christian; the Police and Immigration Office with Christian Brenda.

Guam: the Department of Agriculture, Division of Aquatic and Wildlife Resources with Director Matthew L.G. Sablan, and Assistant Chief Jay Gutierrez; the Division of Management Authority, Branch of Permits with Senior Biologist Anna Barry; the U.S. Fish and Wildlife Service – Office of Law Enforcement with Wildlife Inspector Arthur T. Taimanglo.

Supplementary references

152. Armstrong, E. J. *et al.* Host transcriptomic plasticity and photosymbiotic fidelity underpin *Pocillopora* acclimatization across thermal regimes in the Pacific Ocean. *Nat. Commun.* **14**, 3056 (2023).
153. Voolstra, C. R. *et al.* Resources from: Disparate patterns of genetic divergence in three widespread corals across a pan-pacific environmental gradient highlights species-specific adaptation trajectories. Zenodo https://doi.org/10.5281/ZENODO.7971396 (2022).
154. Porro, B. *et al.* Tara pacific biomarker-based coral phenotype data release: Islands 1 to 10 and 15. Zenodo https://doi.org/10.5281/ZENODO.7148413 (2022).
155. Mokany, K., Ware, C., Woolley, S. N. C., Ferrier, S. & Fitzpatrick, M. C. A working guide to harnessing generalized dissimilarity modelling for biodiversity analysis and conservation assessment. *Glob. Ecol. Biogeogr.* **31**, 802–821 (2022).
156. Washburne, A. D. *et al.* Phylofactorization: A graph partitioning algorithm to identify phylogenetic scales of ecological data. *Ecol. Monogr.* **89**, e01353 (2019).
157. Ide, K. *et al.* Targeted single-cell genomics reveals novel host adaptation strategies of the symbiotic bacteria *Endozoicomonas* in *Acropora tenuis* coral. *Microbiome* **10**, 220 (2022).
158. Pogoreutz, C. *et al.* Coral holobiont cues prime *Endozoicomonas* for a symbiotic lifestyle. *ISME J.* **16**, 1883–1895 (2022).
159. Wang, G. *et al.* Comparative genomics reveal the animal-associated features of the Acanthopleuribacteraceae bacteria, and description of *Sulfidibacter corallicola* gen. nov., sp., nov. *Front. Microbiol.* **13**, 778535 (2022).
160. Fukunaga, Y., Kurahashi, M., Yanagi, K., Yokota, A. & Harayama, S. *Acanthopleuribacter pedis* gen. nov., sp. nov., a marine bacterium isolated from a chiton, and description of Acanthopleuribacteraceae fam. nov., Acanthopleuribacterales ord. nov., Holophagaceae fam. nov., Holophagales ord. nov. and Holophagae classis nov. in the phylum 'Acidobacteria'. *Int. J. Syst. Evol. Microbiol.* **58**, 2597–2601 (2008).

161. Anders, S., Pyl, P. T. & Huber, W. HTSeq—a Python framework to work with high-throughput sequencing data. *Bioinformatics* **31**, 166–169 (2015).
162. Love, M. I., Huber, W. & Anders, S. Moderated estimation of fold change and dispersion for RNA-seq data with DESeq2. *Genome Biol.* **15**, 550 (2014).
163. Blin, K. *et al.* antiSMASH 6.0: Improving cluster detection and comparison capabilities. *Nucleic Acids Res.* **49**, W29–W35 (2021).
164. Wang, M. *et al.* Sharing and community curation of mass spectrometry data with Global Natural Products Social Molecular Networking. *Nat. Biotechnol.* **34**, 828–837 (2016).
165. Blin, K. *et al.* antiSMASH 7.0: New and improved predictions for detection, regulation, chemical structures and visualisation. *Nucleic Acids Res.* **51**, W46–W50 (2023).
166. Johnson, M. *et al.* NCBI BLAST: A better web interface. *Nucleic Acids Res.* **36**, W5–9 (2008).
167. Zallot, R., Oberg, N. & Gerlt, J. A. The EFI web resource for genomic enzymology tools: Leveraging protein, genome, and metagenome databases to discover novel enzymes and metabolic pathways. *Biochemistry* **58**, 4169–4182 (2019).
168. Oberg, N., Zallot, R. & Gerlt, J. A. EFI-EST, EFI-GNT, and EFI-CGFP: Enzyme Function Initiative (EFI) web resource for genomic enzymology tools. *J. Mol. Biol.* **435**, 168018 (2023).
169. Admi, V., Afek, U. & Carmeli, S. Raocyclamides A and B, novel cyclic hexapeptides isolated from the Cyanobacterium *Oscillatoria raoi*. *J. Nat. Prod.* **59**, 396–399 (1996).
170. Tan, L. T., Sitachitta, N. & Gerwick, W. H. The guineamides, novel cyclic depsipeptides from a Papua New Guinea collection of the marine cyanobacterium *Lyngbya majuscula*. *J. Nat. Prod.* **66**, 764–771 (2003).
171. Hayakawa, Y., Sasaki, K., Nagai, K., Shin-ya, K. & Furihata, K. Structure of thioviridamide, a novel apoptosis inducer from *Streptomyces olivoviridis*. *J. Antibiot.* **59**, 6–10 (2006).
172. Sit, C. S., Yoganathan, S. & Vederas, J. C. Biosynthesis of aminovinyl-cysteine-containing peptides and its application in the production of potential drug candidates. *Acc. Chem. Res.* **44**, 261–268 (2011).
173. Mo, T. *et al.* Convergent evolution of the Cys decarboxylases involved in aminovinyl-cysteine (AviCys) biosynthesis. *FEBS Lett.* **593**, 573–580 (2019).
174. Melby, J. O., Nard, N. J. & Mitchell, D. A. Thiazole/oxazole-modified microcins: Complex natural products from ribosomal templates. *Curr. Opin. Chem. Biol.* **15**, 369–378 (2011).
175. Hedlund, B. P. *et al.* SeqCode: A nomenclatural code for prokaryotes described from sequence data. *Nat. Microbiol.* **7**, 1702–1708 (2022).
