# Supplementary material for: Coral microbiomes as reservoirs of unknown genomic and biosynthetic diversity
Source: Nature. 2026 Feb 25;652(8110):686–93. doi: 10.1038/s41586-026-10159-6 (PMC13083261; doi:10.1038/s41586-026-10159-6)
Supplement: Supplementary file 2 — Reporting Summary [file 41586_2026_10159_MOESM2_ESM.pdf]

nature portfolio

Corresponding author(s): Sunagawa, Shinichi

Last updated by author(s): Dec 5, 2025

Reporting Summary

Nature Portfolio wishes to improve the reproducibility of the work that we publish. This form provides structure for consistency and transparency in reporting. For further information on Nature Portfolio policies, see our Editorial Policies and the Editorial Policy Checklist.

Please do not complete any field with "not applicable" or n/a. Refer to the help text for what text to use if an item is not relevant to your study.
For final submission: please carefully check your responses for accuracy; you will not be able to make changes later.

Statistics

For all statistical analyses, confirm that the following items are present in the figure legend, table legend, main text, or Methods section.

n/a	Confirmed	
☐	☒	The exact sample size (*n*) for each experimental group/condition, given as a discrete number and unit of measurement
☐	☒	A statement on whether measurements were taken from distinct samples or whether the same sample was measured repeatedly
☐	☒	The statistical test(s) used AND whether they are one- or two-sided<br>*Only common tests should be described solely by name; describe more complex techniques in the Methods section.*
☐	☒	A description of all covariates tested
☐	☒	A description of any assumptions or corrections, such as tests of normality and adjustment for multiple comparisons
☐	☒	A full description of the statistical parameters including central tendency (e.g. means) or other basic estimates (e.g. regression coefficient) AND variation (e.g. standard deviation) or associated estimates of uncertainty (e.g. confidence intervals)
☐	☒	For null hypothesis testing, the test statistic (e.g. *F*, *t*, *r*) with confidence intervals, effect sizes, degrees of freedom and *P* value noted<br>*Give P values as exact values whenever suitable.*
☒	☐	For Bayesian analysis, information on the choice of priors and Markov chain Monte Carlo settings
☒	☐	For hierarchical and complex designs, identification of the appropriate level for tests and full reporting of outcomes
☒	☐	Estimates of effect sizes (e.g. Cohen's *d*, Pearson's *r*), indicating how they were calculated

Our web collection on statistics for biologists contains articles on many of the points above.

Software and code

Policy information about availability of computer code

Data collection	Data collection is described in detail in Lombard, F. et al. Open science resources from the Tara Pacific expedition across coral reef and surface ocean ecosystems. Sci. Data 10, 324 (2023) and Belser, C. et al. Integrative omics framework for characterization of coral reef ecosystems from the Tara Pacific expedition. Sci. Data 10, 326 (2023).
Data analysis	Open Source/Custom:<br>BBMap (v38.79), metaSPAdes (v3.14.1 or v3.15), BWA (v0.7.17-r1188), MetaBAT 2 (v2.12.1), CheckM (v1.1.3), Anvi'o (v7.1), dRep (v3.0.0), GTDB-Tk (v2.1.0), Prodigal (v2.6.3), Barrnap (v0.9), Aragorn (v1.2.41), fetchMGs (v1.2), antiSMASH (v6.1.1 or v7.1.0), mOTUs (v3.1), CD-HIT (v4.8.1), emapper (v2.1.7), DIAMOND (v2.0.15.153), clust-o-matic, blastn (v2.15.0+), Flye (v2.9.3), AlphaFold (v2.2.0), rtk (v0.93.2), SINA (v1.6.0), MOTHUR (v1.41.0), FastTree (v2.1.11), PhyCA, BBTools (v38.18), HTseq count (v2.0.2), GNPS, and R (v4.2.2-4.3.1) with packages ggplot2 (v3.4.2), tidyverse (v2.0.0), leaflet (v2.1.2), data.table (v1.14.8), ComplexHeatmap (v2.14.0), UpSetR (v1.4.0), ComplexUpset (v1.3.3), ape (v5.7-1), DESeq2 (v1.37.4)<br>The code used for the analyses performed in this study is accessible at GitHub (https://github.com/SushiLab/reef-microbiomics-paper/) and archived on Zenodo (https://zenodo.org/doi/10.5281/zenodo.10201847).<br>Commercial:<br>CytExpert v2.5 (Beckman Coulter), Thermo Xcalibur Qual Browser 4.1 (Thermo Fisher Scientific), Xcalibur Freestyle 1.8 SP2 (Thermo Fisher Scientific), Mnova v12 (Mestrelab Research), TopSpin 3.5/4.1 (Bruker), and Prism 9 (GraphPad)

For manuscripts utilizing custom algorithms or software that are central to the research but not yet described in published literature, software must be made available to editors and reviewers. We strongly encourage code deposition in a community repository (e.g. GitHub). See the Nature Portfolio guidelines for submitting code & software for further information.

Data

Policy information about availability of data

All manuscripts must include a data availability statement. This statement should provide the following information, where applicable:

- Accession codes, unique identifiers, or web links for publicly available datasets
- A description of any restrictions on data availability
- For clinical datasets or third party data, please ensure that the statement adheres to our policy

Tara Pacific short-read metagenomic data generated in this study were submitted to the ENA at the EMBL European Bioinformatics Institute under the Tara Pacific umbrella project PRJEB47249 (accession numbers provided in Supplementary Table 1). Sample provenance and environmental context are available on Zenodo (https://zenodo.org/doi/10.5281/zenodo.4068292). Tara Pacific long-read metagenomic data were submitted to ENA (ERR14224704, ERR14224705, ERR14224706). The publicly available metagenomic data used in this study were downloaded from the ENA, and a summary of their accession numbers is provided in Supplementary Table 1. The MIBiG and BiG-FAM databases can be accessed at https://mibig.secondarymetabolites.org/ and https://bigfam.bioinformatics.nl/, respectively. The reef genomic data used in this study (RMD) are available online (https://microbiomics.io/reef/), and can be interactively explored through the Ocean Microbiomics Database (OMDB; https://omdb.microbiomics.io) as well as contextualised with non-marine environments through the mOTUs online database (https://motus-db.org). All files used for characterising natural products (https://zenodo.org/doi/10.5281/zenodo.14050210) and all other supporting data (https://zenodo.org/doi/10.5281/zenodo.10182966) were deposited on Zenodo.

Research involving human participants, their data, or biological material

Policy information about studies with human participants or human data. See also policy information about sex, gender (identity/presentation), and sexual orientation and race, ethnicity and racism.

Reporting on sex and gender	NA
Reporting on race, ethnicity, or other socially relevant groupings	NA
Population characteristics	NA
Recruitment	NA
Ethics oversight	NA

Note that full information on the approval of the study protocol must also be provided in the manuscript.

Field-specific reporting

Please select the one below that is the best fit for your research. If you are not sure, read the appropriate sections before making your selection.

☐ Life sciences ☐ Behavioural & social sciences ☒ Ecological, evolutionary & environmental sciences

For a reference copy of the document with all sections, see nature.com/documents/nr-reporting-summary-flat.pdf

Ecological, evolutionary & environmental sciences study design

All studies must disclose on these points even when the disclosure is negative.

Study description	This study is a genome-resolved analysis of the coral reef microbiome, promoting coral microbiomes as reservoirs of novel genomic and biosynthetic diversity.
Research sample	Three coral genera were targeted (Millepora spp., Porites spp., Pocillopora spp.) as they are widespread across the Pacific Ocean.
Sampling strategy	Samples were collected from the schooner Tara and by scuba diving as described in Lombard, F. et al. Open science resources from the Tara Pacific expedition across coral reef and surface ocean ecosystems. Sci. Data 10, 324 (2023).
Data collection	Metagenomic data were generated as described in Belser, C. et al. Integrative omics framework for characterization of coral reef ecosystems from the Tara Pacific expedition. Sci. Data 10, 326 (2023). Furthermore, we searched the European Nucleotide Archive and included publicly available coral and sponge metagenomes.
Timing and spatial scale	Samples were collected across the Pacific Ocean during the Tara Pacific expedition from 2016 to 2018.
Data exclusions	No data generated from the Tara Pacific expedition were excluded. For the publicly available metagenomes, we excluded samples for which the metadata was insufficient to clearly identify them as sponge or coral samples.

Reproducibility	Not applicable as this is a field study.
Randomization	Not applicable as this is a field study.
Blinding	Not applicable as this is a field study.

Did the study involve field work? ☒ Yes ☐ No

Field work, collection and transport

Field conditions	All environmental parameters are reported in Pesant, S. et al. Tara Pacific samples provenance and environmental context - version 2. (2020) doi:10.5281/ZENODO.4068292.
Location	99 reefs from 32 islands across the Pacific Ocean.
Access & import/export	Research (UNCLOS) permits<br>Sampling permit for PANAMA under the reference 'SE/AP-18-16' delivered by the Direccion de Areas Protegidas y Vida Silvestre - LIC. Samuel Valdez Diaz Director - Ministerio de Ambiente – Republica de Panama on the 13/06/2016; Sampling permit for PANAMA under the reference '2016-0701-2019-2' delivered by the Smithsonian Tropical Research Institute Instituto Smithsonian de Investigaciones Tropicales - STRI Animal Care and Use Committee (ACUC) on the 28/06/2016; Sampling permit for PANAMA under the reference '2016-0701-2019-2-A1' delivered by the Smithsonian Tropical Research Institute Instituto Smithsonian de Investigaciones Tropicales - STRI Animal Care and Use Committee (ACUC) on the 21/06/2018; Sampling permit for COLOMBIA under the reference 'N°009' delivered by the MINISTERIO DE AMBIENTE Y DESARROLLO SOSTENIBLEPARQUES NACIONALES NATURALES DE COLOMBIA on the 04/03/2016; Sampling permit for CHILE under the reference '13270/24/457/Vrs' delivered by the Servicio Hidrografico y Oceanografico de la Armada de Chile (SHOA) – Patricio Carrasco Hellwig Contraalmirante Director on the 29/08/2016; Sampling permit for UNITED-KINGDOM (PITCAIRN ISLANDS) under the reference 'N/A' delivered by the Government of Pitcairn islands /Environmental, Conservation & Natural Resources Division Manager // Christian Michele on the 25/02/2016; Sampling permit for COOK under the reference '11-16' delivered by the Foundation for National Research – Cook Island Research Committee – Office of the Prime Minister – Elizabeth Wright-Koteka (Chairperson) on the 12/09/2016; Sampling permit for NIUE under the reference '34/16' delivered by the Government of Niue – Office for External Affairs on the 17/11/2016; Sampling permit for SAMOA under the reference 'Memorandum of Agreement' delivered by the THE GOVERNMENT OF THE INDEPENDENT STATE OF SAMOA acting by and through the Ministry of Natural Resources and Environment on the 29/11/2016; Sampling permit for WALLIS AND FUTUNA under the reference 'Arrêté n°2016-527' delivered by the Le Préfet, Administrateur supérieur des îles Wallis et Futuna on the 24/11/2016; Sampling permit for TUVALU under the reference 'MFAT : 449/16' delivered by the Government of Tuvalu – Ministry of Foreign Affairs on the 19/12/2016; Sampling permit for KIRIBATI under the reference '015/16' delivered by the Environment and Conservation Division – Republic of Kiribati on the 24/11/2016; Sampling permit for MICRONESIA under the reference 'Letter' delivered by the Deputy Assistant Secretary – Marine Resources Unit – Department of Resources and Development – Federated States of Micronesia on the 05/04/2017; Sampling permit for GUAM under the reference 'U2021-023' delivered by the Marine Scientific Research Coordinator Office of Ocean and Polar Affairs – United States Department of State Bureau of Oceans and International Environmental and Scientific Affairs on the 27/10/2021; Sampling permit for AMERICAN SAMOA under the reference 'U2021-022' delivered by the Marine Scientific Research Coordinator Office of Ocean and Polar Affairs – United States Department of State Bureau of Oceans and International Environmental and Scientific Affairs on the 27/10/2021; Sampling permit for JAPAN (Tokyo Prefecture; Ogasawara Island) under the reference '28-50' delivered by the Prefecture of Tokyo on the 01/23/2017; Sampling permit for JAPAN (Okinawa Prefecture; Sesoko Island) under the reference '28-74' delivered by the Prefecture of Okinawa on the 04/14/2017; Sampling permit for JAPAN (Japanese EEZ) under the reference 'N/A' delivered by the Ministry of Agriculture, Forestry and Fisheries on the 01/10/2017; Sampling permit for FIJI under the reference '456/2017' delivered by the Ministry of Foreign Affairs – Republic of Fiji on the 11/06/2017; Sampling permit for AUSTRALIA under the reference 'G17/39873.1' delivered by the Great Barrier Reef Marine Park Authority and Department of Foreign Affairs and Trade on the 30/08/2017; Sampling permit for NEW-CALEDONIA (SOUTH PROVINCIA) under the reference 'Arrêté n°2720-2017/ARR/DENV modifiant l'arrêté 1515-2017/ARR/DENV du 04 août 2017' delivered by the Président de l'Assemblée de la Province Sud de la Nouvelle-Calédonie on the 06/09/2017; Sampling permit for NEW-CALEDONIA (CHESTERFIELD) under the reference 'Arrêté n°2017-2069/GNC' delivered by the Haut-Commissariat de la République en Nouvelle-Calédonie – Gouvernement de Nouvelle-Calédonie – République Française on the 29/08/2017; Sampling permit for SOLOMON ISLANDS under the reference 'Form 01' delivered by the Solomon Islands Maritime Safety Administration on the 20/09/2017; Sampling permit for PAPUA NEW-GUINEA under the reference '907/2017 (diplomatic clearance n°0232)' delivered by the Department of Foreign Affairs and Trade of the Independent State of Papua New Guinea on the 27/10/2017; Sampling permit for PALAU under the reference 'RE-18-04' delivered by the Ministry of Natural Resources, Environment and Tourism – Republic of Palau on the 21/12/2017; Sampling permit for CHINA (HONG-KONG) under the reference 'CMO-N00811' delivered by the Marine Department, Hong-Kong, China on the 15/03/2018; Sampling permit for TAIWAN (Pingtung county) under the reference '10707821600' delivered by the Pingtung Agri-Fish; National Taiwan Ocean University on the 06/04/2018; Sampling permit for TAIWAN (Taitung county) under the reference '1070033041' delivered by the Taitung Agri-Fish; National Taiwan Ocean University on the 12/02/2018; Sampling permit for USA (HAWAII) under the reference 'U2018-010' delivered by the United States Department of State Bureau of Oceans and International Environmental and Scientific Affairs on the 06/06/2018; Sampling permit for MEXICO under the reference 'PPF/DGOPA-291/17' delivered by the Secrataria de Agricultura, Ganaderia, Desarrollo rural, pesca y alimentacion – Comision Nacional de Acuacultura y Pesca – Direccion General de Ordenamiento Pesquero y Acuicola – Estados Unidos Mexicanos on the 28/08/2018; Sampling permit for CLIPPERTON under the reference 'HC/1195/CAB' delivered by the Haut-Commissariat de la République Polynésie Française on the 13/06/2018; Sampling permit for COSTA RICA under the reference M-C-SINAC-PNI-SE-002-2022 delivered by the Sistema Nacional de Áreas de Conservación (SINAC) on the 29/09/2022; Sampling permit for USA (MAINLAND) under the reference 'U2018-010' delivered by the United States Department of State Bureau of Oceans and International Environmental and Scientific Affairs on the 06/06/2018; Sampling permit for CANADA under the reference 'Letter of regularization' delivered by the Sécurité et relations de défense (IGR)/Affaires mondiales Canada on the 05/01/2022; Sampling permit for NEW-ZEALAND under the reference 'Letter of regularization' delivered by the Ministry of Foreign Affairs and Trade on the

05/10/2021; Sampling permit for IRELAND under the reference '572/22' delivered by the Department of Foreign Affairs on the 09/06/2022.

CITES permits
CITES export permit for PANAMA (I01) under the reference 'SEX/A-72-16' delivered by the Autoridad Nacional del Ambiente (ANAM) de la República de Panamá – Autoridad Administrativa CITES on the 28/07/2016; CITES final import permit under the reference 'FR1609100066-I' delivered the 04/08/2016 by the DRIEE ILE-DE-FRANCE; CITES export permit for PANAMA (I02) under the reference 'SEX/A-72-16' delivered by the Autoridad Nacional del Ambiente (ANAM) de la República de Panamá – Autoridad Administrativa CITES on the 28/07/2016; CITES final import permit under the reference 'FR1609100066-I' delivered the 04/08/2016 by the DRIEE ILE-DE-FRANCE; CITES export permit for PANAMA (I31) under the reference 'SEX/APO-1-2018' delivered by the Ministerio de Ambiente on the 30/08/2018; CITES final import permit under the reference 'FR1807523129-I' delivered the 19/10/2018 by the DRIEE ILE-DE-FRANCE; CITES export permit for PANAMA (I32) under the reference 'SEX/APO-1-2018' delivered by the Ministerio de Ambiente on the 30/08/2018; CITES final import permit under the reference 'FR1807523129-I' delivered the 19/10/2018 by the DRIEE ILE-DE-FRANCE; CITES export permit for COLOMBIA (I03) under the reference '41499' delivered by the Ministerio de Ambiente y Desarrollo Sostenible de la República de Colombia on the 13/02/2017; CITES final import permit under the reference 'FR1707506158-I' delivered the 17/03/2017 by the DRIEE ILE-DE-FRANCE; CITES export permit for CHILE (I04) under the reference '16CL000007WS' delivered by the Servicio Nacional de Pesca y Acuicultura on the 02/09/2016; CITES final import permit under the reference 'FR1607525599-I' delivered the 03/11/2016 by the DRIEE ILE-DE-FRANCE; CITES export permit for UNITED-KINGDOM (PITCAIRN ISLANDS; I05) under the reference 'FR1698700198-E' delivered by the Haut-Commissariat de la République en Polynésie Française on the 03/11/2016; CITES final import permit under the reference 'FR1607525646-I' delivered the 04/11/2016 by the DRIEE ILE-DE-FRANCE; CITES export permit for FRENCH POLYNESIA (GAMBIER – TUAMOTU; I06) under the reference 'FR1698700198-E' delivered by the Haut-Commissariat de la République en Polynésie Française on the 03/11/2016; CITES final import permit under the reference 'FR1607525646-I' delivered the 04/11/2016 by the DRIEE ILE-DE-FRANCE; CITES export permit for MOOREA (I07) under the reference 'FR1698700218-E' delivered by the Haut-Commissariat de la République en Polynésie Française on the 21/11/2016; CITES final import permit under the reference 'FR1707503441-I ' delivered the 07/02/2017 by the DRIEE ILE-DE-FRANCE; CITES export permit for COOK (I08) under the reference 'CK/2016 – 14278' delivered by the Tu'anga Taporoporo national environment service of the Cook Islands on the 17/11/2016; CITES final import permit under the reference 'FR1707503442-I ' delivered the 07/02/2017 by the DRIEE ILE-DE-FRANCE; CITES export permit for NIUE (I09) under the reference 'N/A' delivered by the N/A on the N/A; CITES final import permit under the reference 'FR1707511900-I' delivered the 11/06/2017 by the DRIEE ILE-DE-FRANCE; CITES export permit for SAMOA (I10) under the reference 'SAMC16012' delivered by the Ministry of Natural Resources and Environment (MNRE) of the Government of Samoa on the 29/11/2016; CITES final import permit under the reference 'FR1707503440-I' delivered the 07/02/2017 by the DRIEE ILE-DE-FRANCE; CITES export permit for WALLIS AND FUTUNA (I11) under the reference 'WF/C/16/01' delivered by the Préfet – Administrateur Supérieur – Chef du territoire des Îles Wallis et Futuna on the 25/12/2016; CITES final import permit under the reference 'FR1707503441-I ' delivered the 07/02/2017 by the DRIEE ILE-DE-FRANCE; CITES export permit for TUVALU (I12) under the reference '1204 (Quarantine document)' delivered by the Plant Protection and Quarantine Services - Ministry of Natural Resources - TUVALU GOVERNMENT on the 03/01/2017; CITES final import permit under the reference 'FR1707511900-I' delivered the 11/06/2017 by the DRIEE ILE-DE-FRANCE; CITES export permit for KIRIBATI (I13) under the reference '015/16 (UNCLOS permit)' delivered by the Fisheries Division, Ministry of Fisheries & Marine Resources Development -- GOVERNMENT OF KIRIBATI on the 12/01/2017; CITES final import permit under the reference 'FR1707511900-I' delivered the 11/06/2017 by the DRIEE ILE-DE-FRANCE; CITES export permit for MICRONESIA (CHUUK; I14) under the reference 'CFM17-01-01' delivered by the Department of Resources and Development – Division of Resource Management and Development – Office of Marine Resources on the 19/01/2017; CITES final import permit under the reference 'FR1707511900-I' delivered the 11/06/2017 by the DRIEE ILE-DE-FRANCE; CITES export permit for GUAM (I15) under the reference '17US18844C/9' delivered by the U.S. Fish and Wildlife service – Division of management authority – Branch of permits on the 02/03/2017; CITES final import permit under the reference 'FR1707503440-I' delivered the 07/02/2017 by the DRIEE ILE-DE-FRANCE; CITES export permit for JAPAN (OGASAWARA; I16) under the reference '17JP001279/TE' delivered by the Trade and Economic Cooperation Bureau – Ministry of Economy, Trade and Industry (METI) on the 16/05/2017; CITES final import permit under the reference 'FR1707511899-I' delivered the 06/06/2017 by the DRIEE ILE-DE-FRANCE; CITES export permit for JAPAN (SESOKO; I17) under the reference '17JP001280/TE' delivered by the Trade and Economic Cooperation Bureau – Ministry of Economy, Trade and Industry (METI) on the 16/05/2017; CITES final import permit under the reference ' FR1707511898-I ' delivered the 06/06/2017 by the DRIEE ILE-DE-FRANCE; CITES export permit for FIJI (I18) under the reference 'FJ/EXP-03055' delivered by the Fisheries Department - Government of Fiji on the 08/06/2017; CITES final import permit under the reference 'FR1707521006-I' delivered the 27/09/2017 by the DRIEE ILE-DE-FRANCE; CITES export permit for AUSTRALIA (I19) under the reference 'PWS2017-AU-001613 ' delivered by the Department of the Environment and Energy of the Australian Government on the 22/08/2017; CITES final import permit under the reference 'FR1707521097-I' delivered the 29/09/2017 by the DRIEE ILE-DE-FRANCE; CITES export permit for NEW-CALEDONIA (SOUTH PROVINCIA; I21) under the reference 'FR1798800075-E' delivered by the Haut-Commissariat de la République en Nouvelle-Calédonie / DAFE on the 22/09/2017; CITES final import permit under the reference 'FR1707521096-I' delivered the 29/09/2017 by the DRIEE ILE-DE-FRANCE; CITES export permit for NEW-CALEDONIA (CHESTERFIELD; I20) under the reference 'FR1798800075-E' delivered by the Haut-Commissariat de la République en Nouvelle-Calédonie / DAFE on the 22/09/2017; CITES final import permit under the reference 'FR1707521096-I' delivered the 29/09/2017 by the DRIEE ILE-DE-FRANCE; CITES export permit for SOLOMON ISLANDS (I22) under the reference 'EX2017/188' delivered by the Ministry of Environment, Climate Change, Disaster Management and Met on the 19/10/2017; CITES final import permit under the reference 'FR1807501178-I' delivered the 15/01/2018 by the DRIEE ILE-DE-FRANCE; CITES export permit for PAPUA NEW-GUINEA (I23) under the reference '18004' delivered by the Conservation and Environment Protection Authority (CEPA) on the 06/12/2017; CITES final import permit under the reference 'FR1807508641-I' delivered the 24/04/2018 by the DRIEE ILE-DE-FRANCE; CITES export permit for PAPUA NEW-GUINEA (I24) under the reference '18004' delivered by the Conservation and Environment Protection Authority (CEPA) on the 06/12/2017; CITES final import permit under the reference 'FR1807508641-I' delivered the 24/04/2018 by the DRIEE ILE-DE-FRANCE; CITES export permit for PALAU (transect; I25) under the reference 'PW18-004' delivered by the Office of the Minister – Ministry of Natural Resources, Environment and Tourism on the 10/01/2018; CITES final import permit under the reference 'FR1807501177-I' delivered the 16/01/2018 by the DRIEE ILE-DE-FRANCE; CITES export permit for PALAU (leg; I25) under the reference 'PW18-009' delivered by the Office of the Minister – Ministry of Natural Resources, Environment and Tourism on the 01/10/2018; CITES final import permit under the reference 'FR1807512823-I' delivered the 13/06/2018 by the DRIEE ILE-DE-FRANCE; CITES export permit for CHINA (HONG-KONG; I26) under the reference 'APO/EL 3/18' delivered by the Agriculture, Fisheries and Conservation Department of Hong-Kong Special Administrative Region on the 16/04/2018; CITES final import permit under the reference 'FR1807508518-I' delivered the 23/04/2018 by the DRIEE ILE-DE-FRANCE; CITES export permit for TAIWAN (I27) under the reference 'FTS507W0147330' delivered by the Bureau of Foreign Trade – Ministry of Economic Affairs on the 21/06/2018; CITES final import permit under the reference 'FR1807515565-I' delivered the 18/07/2018 by the DRIEE

ILE-DE-FRANCE; CITES export permit for USA (HAWAII; I28) under the reference '18US97917C/9' delivered by the U.S. Fish and Wildlife service – Division of management authority – Branch of permits on the 26/07/2018, CITES final import permit under the reference 'FR180751766-I' delivered the 09/08/2018 by the DRIEE ILE-DE-FRANCE.

Disturbance

Contact with corals was minimised.

Reporting for specific materials, systems and methods

We require information from authors about some types of materials, experimental systems and methods used in many studies. Here, indicate whether each material, system or method listed is relevant to your study. If you are not sure if a list item applies to your research, read the appropriate section before selecting a response.

Materials & experimental systems

n/a	Involved in the study
☒	☐ Antibodies
☒	☐ Eukaryotic cell lines
☒	☐ Palaeontology and archaeology
☒	☐ Animals and other organisms
☒	☐ Clinical data
☒	☐ Dual use research of concern
☒	☐ Plants

Methods

n/a	Involved in the study
☒	☐ ChIP-seq
☒	☐ Flow cytometry
☒	☐ MRI-based neuroimaging

Plants

Seed stocks

NA

Novel plant genotypes

NA

Authentication

NA

This checklist template is licensed under a Creative Commons Attribution 4.0 International License, which permits use, sharing, adaptation, distribution and reproduction in any medium or format, as long as you give appropriate credit to the original author(s) and the source, provide a link to the Creative Commons license, and indicate if changes were made. The images or other third party material in this article are included in the article's Creative Commons license, unless indicated otherwise in a credit line to the material. If material is not included in the article's Creative Commons license and your intended use is not permitted by statutory regulation or exceeds the permitted use, you will need to obtain permission directly from the copyright holder. To view a copy of this license, visit http://creativecommons.org/licenses/by/4.0/
