# Supplementary material for: Coral microbiomes as reservoirs of unknown genomic and biosynthetic diversity
Source: Nature. 2026 Feb 25;652(8110):686–93. doi: 10.1038/s41586-026-10159-6 (PMC13083261; doi:10.1038/s41586-026-10159-6)
Supplement: Supplementary file 3 — Supplementary Tables 1–12. [file 41586_2026_10159_MOESM3_ESM.zip › 2023-11-20683E-s3/Supplementary-Table-legends.docx]

### File name

supplementary-table-1.xlsx

### Title

Supplementary Table 1: *Tara* Pacific metagenomes

### Summary

Sheet 1: Coral metagenomes; Sheet 2: Seawater metagenomes; Sheet 3: Long-read metagenomes.

### File name

supplementary-table-2.xlsx

### Title

Supplementary Table 2: Publicly available metagenomes from coral and sponge studies

### Summary

Sheet 1: Coral and sponge metagenomic studies included; Sheet 2: Metagenomic samples summary; Sheet 3: Previously reported MAGs from the publicly available coral metagenomes.

### File name

supplementary-table-3.xlsx

### Title

Supplementary Table 3: Reef Microbiomics Database (RMD) summary

### Summary

Sheet 1: Description of the RMD by dataset source; Sheet 2: Description of the RMD by coral group; Sheet 3: Phylogenomic novelty (unique to source indicated in parenthesis); Sheet 4: Number of different host coral genera from which genomes were reconstructed per microbial species; Sheet 5: Genome summary.

### File name

supplementary-table-4.xlsx

### Title

Supplementary Table 4: Publicly available isolate genomes from coral and sponge studies

### Summary

Sheet 1: Isolate genome studies included; Sheet 2: Isolate genome summary.

### File name

supplementary-table-5.xlsx

### Title

Supplementary Table 5: Biosynthetic potential of the RMD genomes

### Summary

Sheet 1: antiSMASH summary for all genomes in the RMD; Sheet 2: Biosynthetic gene-cluster families (GCFs) for the RMD clustered with the OMD and MIBiG; Sheet 3: Natural product class comparison between the RMD and OMD; Sheet 4: Natural product class comparison across coral groups and sponges.

### File name

supplementary-table-6.xlsx

### Title

Supplementary Table 6: Natural products isolated from corals

### Summary

Sheet 1: Summary of natural products isolated from any of the 15 coral genera for which we predicted microbially encoded BGCs.

### File name

supplementary-table-7.xlsx

### Title

Supplementary Table 7: Candidate BGC-rich lineages

### Summary

Sheet 1: Summary of the candidate BGC-rich MAGs identified in the RMD.

### File name

supplementary-table-8.xlsx

### Title

Supplementary Table 8: Coral host transcriptomics and generalised dissimilarity models

### Summary

Sheet 1: Map between the sample names across data type; Sheet 2: Environmental and biomarker variables used in the models.

### File name

supplementary-table-9.xlsx

### Title

Supplementary Table 9: *aci* cluster

### Summary

Sheet 1: DNA sequences for *aci* cluster; Sheet 2: Protein sequences for *aci* cluster; Sheet 3: Exact masses MS1; Sheet 4: MS2 fragments.

### File name

supplementary-table-10.xlsx

### Title

Supplementary Table 10: *tha* cluster

### Summary

Sheet 1: DNA sequences for *tha* cluster; Sheet 2: Protein sequences for *tha* cluster; Sheet 3: Exact masses MS1 labelled medium; Sheet 4: MS2 fragments native; Sheet 5: MS2 fragments point mutants; Sheet 6: Neutrophil elastase inhibition.

### File name

supplementary-table-11.xlsx

### Title

Supplementary Table 11: *the* cluster

### Summary

Sheet 1: DNA sequences for *the* cluster; Sheet 2: Protein sequences for *the* cluster; Sheet 3: Exact masses MS1; Sheet 4: MS2 fragments native; Sheet 5: MS2 fragments truncations; Sheet 6: MS2 fragments PG-1 fusion.

### File name

supplementary-table-12.xlsx

### Title

Supplementary Table 12: Summary of the sampling permits

### Summary

Sheet 1: Information on the sampling permits and restrictions regarding commercial use.
